# Exposure to elevated temperature during development affects bumblebee foraging behavior

**DOI:** 10.1093/beheco/arac045

**Published:** 2022-06-03

**Authors:** Maxence Gérard, Bérénice Cariou, Maxime Henrion, Charlotte Descamps, Emily Baird

**Affiliations:** INSECT Lab, Division of Functional Morphology, Department of Zoology, Stockholm University, Svante Arrhenius väg 18b, 11418 Stockholm, Sweden; INSECT Lab, Division of Functional Morphology, Department of Zoology, Stockholm University, Svante Arrhenius väg 18b, 11418 Stockholm, Sweden; Sorbonne Université, Faculté des Sciences et Ingénierie, 5 place Jussieu, 75005 Paris, France; INSECT Lab, Division of Functional Morphology, Department of Zoology, Stockholm University, Svante Arrhenius väg 18b, 11418 Stockholm, Sweden; Ecole Normale Supérieure de Lyon, 15 parvis René Descartes, Lyon, France, and; Earth and Life Institute-Agrotnomy, UCLouvain, Croix du Sud 2, box L7.05.14, 1348 Louvain-la-Neuve, Belgium; INSECT Lab, Division of Functional Morphology, Department of Zoology, Stockholm University, Svante Arrhenius väg 18b, 11418 Stockholm, Sweden

**Keywords:** body size, *Bombus terrestris*, colony development, foraging behavior, geometric morphometrics, global warming

## Abstract

Bee foraging behavior provides a pollination service that has both ecological and economic benefits. However, bee population decline could directly affect the efficiency of this interaction. Among the drivers of this decline, global warming has been implicated as an emerging threat but exactly how increasing temperatures affect bee foraging behavior remains unexplored. Here, we assessed how exposure to elevated temperatures during development affects the foraging behavior and morphology of workers from commercial and wild *Bombus terrestris* colonies. Workers reared at 33 °C had a higher visiting rate and shorter visiting time than those reared at 27°C. In addition, far fewer workers reared at 33 °C engaged in foraging activities and this is potentially related to the drastic reduction in the number of individuals produced in colonies exposed to 33 °C. The impact of elevated developmental temperature on wild colonies was even stronger as none of the workers from these colonies performed any foraging trips. We also found that rearing temperature affected wing size and shape. Our results provide the first evidence that colony temperature can have striking effects on bumblebee foraging behavior. Of particular importance is the drastic reduction in the number of workers performing foraging trips, and the total number of foraging trips made by workers reared in high temperatures. Further studies should explore if, ultimately, these observed effects of exposure to elevated temperature during development lead to a reduction in pollination efficiency.

## Teaser

Climate change is increasingly linked to wild bee loss though the impact on their behavior is poorly documented. Using an experimental approach, we show that elevated developmental temperatures can have drastic effects on bumblebee foraging behavior. Workers developed at elevated temperatures performed fewer foraging trips, potentially because of changes in wing morphology and lower worker production. Ultimately, this may lead to fitness costs for the colony and affect pollination efficiency.

## INTRODUCTION

Plant–pollinator interactions are crucial from both ecological and economic perspectives. Indeed, around 85% of flowering plants depend on animals to carry pollen from anthers to stigma of the flowers, thus allowing the reproduction of the plant (Ollerton et al. 2011). Most of these pollinators are insects, which provide an important ecosystem service both for agricultural production and food security by facilitating plant sexual reproduction (IPBES 2016). However, pollinators and their pollination services are threatened by human activities, limiting plant reproductive success (Thomann et al. 2013). The decline of pollinators can be associated with different drivers such as (1) habitat changes and the decrease of floral resources, (2) climate change, (3) misuse/overuse of agrochemicals, and (4) introduction of exotic/managed species and their associated pathogens (Potts et al. 2016; Klein et al. 2018; Dicks et al. 2020). Among them, climate change and global warming represent an emerging threat for some groups (but see Ghisbain et al., 2021), notably with the increase of heatwaves, as well as the overall increase of annual average temperature (IPCC 2019). Higher temperatures are known to affect the physiology (Scaven and Rafferty 2013), phenology (Pyke et al. 2016; Duchenne et al. 2020), responses to sensory stimuli (Perl et al. 2022), morphology (Gérard et al. 2018a), and distribution of pollinators (Sirois-Delisle and Kerr 2018), such as bees, leading to potential mismatches with the plants on which they forage (Hegland et al. 2009; Gérard et al. 2020) and contributing to the widespread decline of species (Soroye et al. 2020).

Among bees, developmental temperature can impact different life cycle parameters. High temperatures decrease development time (Tepedino and Parker 1986; Radmacher and Strohm 2010), increase mortality before emergence (O’Neill et al. 2011), and affect colony productivity and longevity (Weidenmüller et al. 2002; Holland and Bourke 2015). In addition, elevated temperatures can affect morphological traits. For example, the size and shape of male bumblebee wings are affected by stressful rearing temperatures (Gérard et al. 2018a) and increasing environmental temperatures have been linked to a decrease in tongue and body size in workers and queens (Miller-Struttman et al. 2015; Gérard et al. 2018b), although opposite results also exist (Gérard et al. 2021). However, it is not clear if high developmental temperature could impact the foraging behavior of worker bees, and if changes in morphology and colony production may explain changes in the foraging behavior.

A plastic response, known as the temperature-size rule (TSR), describes the reduction in adult body size in response to increased developmental temperature, a phenomenon found in most insects (Atkinson 1994; Angilletta and Dunham 2003). A temperature-related reduction in body size could directly affect foraging behavior as smaller body sizes are associated with reduced foraging range (Greenleaf et al. 2007; Kendall et al. 2019) and foraging rate in bumblebees (Spaethe and Weidenmüller 2002). Recent studies also show that, while global warming has led to a reduction in the tongue length of bees, this reduction has not been matched by a reduction in corolla depth in co-occurring flowers (Miller-Struttmann et al. 2015; Christmas et al. 2021). This is the first evidence that temperature-related morphological mismatches in plant–pollinator interactions are already occurring.

Temperature-related morphological changes are likely to strongly affect the foraging behavior of bees. Indeed, flight capacity, which is crucial for pollination, is directly impacted by wing and body size. For example, shorter and broader wings are more efficient for maneuverability, while long and slender wings are more efficient for long distance flight and dispersal (Betts and Wooton 1988; DeVries et al. 2010). Modifications of the wings are even known to affect the time that bees take during each foraging bout (Foster and Cartar 2011). Other important parameters related to pollination, like visiting rate (i.e., number of flowers a pollinator visits per unit of time) or the number of foraging trips, may also be impacted by these morphological changes, although this remains poorly studied.

Despite the known impacts of elevated temperatures on bee colony development and morphology, the functional consequences of these changes on pollination behavior are unknown as these variables have never been assessed together on the same colonies. In addition, most of the studies in controlled conditions have been conducted on bumblebee colonies developed on a large scale for commercial purposes (defined here as *commercial* colonies), and it remains unclear how wild colonies react to temperature stress (Velthuis and van Doorn 2006; Gérard et al. 2018a). To begin to address this knowledge gap, we used commercial and wild bumblebee colonies to investigate the impact of high developmental temperatures on bumblebee foraging behavior, morphology and colony development. We reared both commercial colonies and colonies from wild-caught queens of the buff-tailed *Bombus terrestris* L. (1758) at an optimal rearing temperature of 27 °C (Röseler 1985; Vogt 1986) and at a stressful temperature of 33 °C, which is above the set-point at which bumblebees start fanning (Vogt 1986, Weidenmüller et al. 2002). The foraging behavior of workers from these colonies was measured using two plant species: *Borago officinalis* L. (1753) and *Campanula persicifolia* L. (1753). We also measured different morphological traits that are important for foraging and the number of workers produced. When possible, we measured the foraging parameters on the same individuals as the ones used for analysis of morphological traits. We hypothesized that high developmental temperatures have a negative impact on foraging behavior, that this may be linked to changes in morphological features or worker production and that these effects may differ between commercial and wild colonies. Through selective breeding, commercial bumblebee colonies may be better at buffering stressors, while wild colonies could be more vulnerable to rapid changes. For example, commercial breeders likely favor queens that produce a larger number of individuals with larger body sizes, which may increase the robustness of both individuals and the colony to stressors. However, the opposite may also be true: commercial colonies may be worse at buffering environmental stressors, since selection for optimal performance may have removed genetic variation from these colonies that would be beneficial in fluctuating or unpredictable environments, making commercial colonies more vulnerable to rapid changes than wild ones.

## MATERIALS AND METHODS

### Bumblebee rearing

Bumblebee rearing and plant–pollinator experiments were conducted at the Tovetorp Zoological Research Station of Stockholm University (Sweden). We reared four “*commercial”* colonies of *Bombus terrestris* from Koppert Biological Systems (Berkel en Rodenrijs, The Netherlands) and four “*wild”* colonies from wild queens of *B. terrestris*, caught in Östergötland and Uppland (Sweden). The colonies were reared in 28 cm × 25 cm × 20 cm plastic boxes. Two commercial colonies and two wild colonies were placed in one of two rooms (i.e., four colonies per room), one kept at 27 °C and one kept at 33 °C. While 27 °C is an optimal temperature for bumblebee development (Röseler 1985; Vogt 1986), 33 °C represents an ecologically relevant stressful temperature that bumblebees can experience while foraging (Couvillon et al. 2010), as well as in their nest under the ground (Grad and Gradisek 2018), and is expected to occur regularly during summer in the distribution range of many species (Rasmont et al. 2015; IPCC 2021). The colonies were kept in the dark with both rooms having a humidity range of 30%-40%. Bumblebees were fed ad libitum with the Koppert Natupol Smart sugar solution and fresh-frozen organic pollen (Naturprodukter, Raspowder Bipollen). The colony numbers were limited to four per treatment because it was not possible to adequately maintain more while keeping track of individuals. In addition, the flight room could only accommodate 4 colonies at a time.

Each colony contained 25 workers, one queen and existing brood on the first day of the experiment. After 25 days of development, which corresponds to the duration of the development from egg to adult, all individuals in each colony were marked (Duchateau and Velthuis 1988). On day 26, any newly emerged workers had experienced the full temperature treatment during development, and these were individually marked with a unique numbered and colored dot glued on the thorax (beekeeper glue, odorless, and non-toxic). This individual marking also allowed the discrimination among colonies because each colony was assigned a particular color. On day 32, the colonies were placed in the flight room to conduct the plant–pollinator experiments. The sugar solution and pollen were removed from the colonies two days before the start of the experiments to encourage workers to leave the colony and forage.

### Plant–pollinator interaction

For the plant–pollinator interaction experiments, we selected two plant species: *Borago officinalis* and *Campanula persicifolia*. These two species are known to be attractive for bumblebees, are widespread in Europe (Carreck and Williams 2002; Samnegard et al. 2011; Descamps et al. 2018), and have separated flowers, which is necessary for accurately measuring the foraging parameters. The plants were bought a few days before the experiments started from a commercial nursery (Slottsträdgården Ulriksdal Solna, Sweden), where they grew in controlled conditions. Before the experiments, the plants were kept in a controlled room at 22 °C with 16 h light per day and were watered daily.

The foraging experiments were conducted in a 6 m × 6 m × 3 m free-flight room at a constant temperature, luminosity, and relative humidity (25 °C, 2050 lx and 21%, respectively) under 14:10 h light/dark cycle. Colonies from the same temperature treatment (*n* = 4 per treatment) were placed 46 cm apart on a 60 cm high table 4 m from the plant pots. A plastic tube, 5 cm long and 1 cm in diameter linked the colony entrance to a landing platform. Microscope cameras (Digital Microscope, China) were placed above the tube and connected to a computer so that all entrances and exits of the bees were continuously recorded. Colonies were left for a day in the flight room to allow the bees to acclimatize to the room and the flowers. The foraging parameters were measured during the following 3 days with eight pots of *C. persicifolia*, followed by a further 3 days of measuring with eight pots of *B. officinalis*. The same procedure was repeated for colonies from each temperature treatment.

Several foraging parameters were measured—visiting time, visiting rate, number of foraging trips per worker, total number of foraging trips per treatment, total number of workers performing the foraging trips per treatment and foraging time. These parameters were only measured on workers that experienced the full temperature treatment during their development. Visiting time was defined as the time passed from the first contact with one particular flower to the last contact with this same flower; the data for a same individual for each day and for a same plant pot were aggregated and averaged. Visiting rate was defined as the number of flowers visited by a worker within 1 min; the data for a same individual for each day were aggregated and averaged. These two parameters were measured by following the workers with a chronometer during 5 min periods (this was done by only one experimenter at a time), repeated every 20 min from 9 a.m. to 7 p.m. during a total of 12 days. We removed the extreme points (i.e., values above upper quartile 3 + 3 × interquartile range or below lower quartile Q1 – 3 × interquartile range) from the visiting time and rate data, as they correspond to biologically non-relevant data (i.e., bumblebees landing on a flower, starting to gather the pollen then staying on the flower for a long time). The number of foraging trips was defined as the number of times an individual left and returned to the colony; the data for a same individual for each day were aggregated and averaged. The total number of workers performing the foraging trips per treatment was also calculated as well as the total number of foraging trips per treatment; these two variables were aggregated for the whole experiment. Finally, we measured foraging time, defined as the time between the exit and the return of a worker into the colony; the data for a same individual were not aggregated. These last four parameters were measured using videos registered with the software OBS (https://obsproject.com/fr) 8 h per day. Individuals that entered the wrong colony were not counted.

To assess the impact of rearing temperature and colony origin (i.e., *commercial* or *wild*) on the measured foraging parameters, we built generalized linear mixed models (GLMM) using the lmer4 R package, computed with R statistics (R Core Team 2020). The trips of bees that left the colony but that did not visit flowers were not included in the analysis. Overall, only 19% of flights included flower visits and were therefore considered in the analysis (note that no foraging flights were observed in the 33 °C treatment for the wild colonies). We used a Gamma distribution for the foraging time, visiting time and visiting rate, when the assumption of residuals normality was not met (even when using log or rank transformation). A Poisson distribution, appropriate for count data, was used for the number of foraging trips. To avoid for potential effects of floral morphology, we ran separate models for the two plant species. In the models, foraging parameters were fitted as the response variable, temperature, colony origin and their interaction were fitted as fixed effects, and colony ID (the individual identifier of each colony from 1 to 8) as well as individual ID (nested in colony ID) were fitted as random effects. Including individual ID as a random effect was necessary to account for pseudoreplication. When a random variable was explaining less than 0.01% of the variance that remained in the residuals, we removed it from the model. If the colony origin or the interaction between temperature and colony origin were not significant, we also calculated models by removing these fixed variables and selected the best model using the lowest AICc.

### Morphological traits

At the end of the experiment, 25 workers of each colony (when possible) were collected to obtain morphological data (Supplementary Table S1 for the total dataset per colony). All these workers were exposed to either 27 °C or 33 °C throughout their development. The inter-tegular distance (ITD; the distance between the two insertion points of the wings) was measured as a proxy for bumblebee body size (Cane 1987), using a digital caliper (Cocraft, Insjön, Sweden). Proboscis, forewings, and antennae were removed and photographed using a microscope (Leica Wild M3Z, Wetzlar, Germany) coupled to a camera (Canon EOS 70D, Tokyo, Japan). Right antenna and proboscis length were measured using ImageJ (Schneider et al. 2012), including the flagellum and the pedicel for the antennae and the glossa for the proboscis. The shape of wings was characterized using two-dimensional Cartesian coordinates of 18 landmarks using tps-DIG v2.32 (Rohlf 2010) on each digitized forewing. We then used the Generalized Procrustes Analysis (i.e., gpagen function; package geomorph, Adams and Otarola-Castillo 2013) to superimpose the landmark configurations, which removes all the non-shape components of the wing by scaling, translating and rotating each wing. The centroid size of each wing, which can be used as a proxy for wing size (Gérard et al. 2018b), was calculated from the square root of the sum of squared distance between each landmark and the centroid of a landmark configuration (Bookstein 1991). To assess the impact of rearing temperature and colony origin on the morphological traits, we built GLMM using the lmer4 R package, computed with R statistics (R Core Team, 2020). When the assumption of residual normality (using the function shapiro.test) was not met, even using log or rank transformation, we used a Gamma distribution which is appropriate for continuous positive data. In the models, morphological trait sizes were fitted as response variables, temperature, colony origin and their interaction as fixed effects, and colony ID as a random effect. The only morphological traits for which the condition of normality was met were the ITD and antennal length; the drivers of the two other morphological traits have thus been assessed using generalized models with a Gamma distribution. For wing shape, we used a Procrustes ANOVA which allowed us to include a multidimensional response variable (procD.lm function; package geomorph, Adams and Otarola-Castillo 2013). We also used a Principal Component Analysis to visualize the wing shape results, using the function gm.prcomp (geomorph R package).

### Colony development

To investigate for a potential impact of developmental temperatures on colony production, we recorded the total number of individuals from each caste (worker, queen, and male) that had been produced by each colony (8 colonies in total) from the day we set up the colonies in the temperature-controlled rooms (i.e., total number of individuals that died during the experiment, as well as those that were still alive at the end of the experiment).

## RESULTS

### Foraging behavior—visiting time

**Figure 1:**
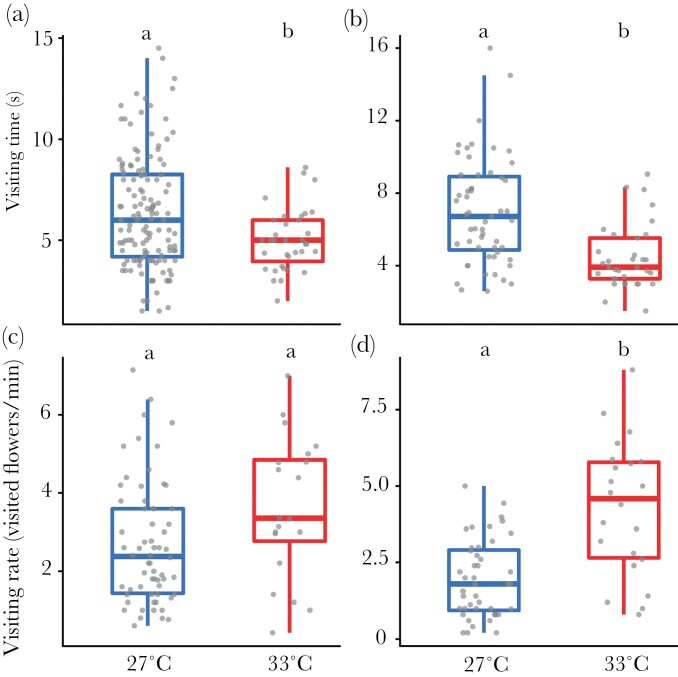
Impact of developmental temperature on bumblebee foraging behavior. (A) Visiting time (s) for *Borago* (*n* = 169). (B) Visiting time (s) for *Campanula* (*n* = 88). (C) Visiting rate (visited flowers per minute) for *Borago* (*n* = 82). (D) Visiting rate (visited flowers per minute) for *Campanula* (*n* = 66). Letters at the top of the boxplots indicate significant differences when the letters are different.

The model that best explained the variation in visiting time of *Borago officinalis* (*n* = 169) included temperature treatment and colony origin as fixed factors (next best model ΔAICc 0.6, Supplementary Table S2 for details of the model). However, this was not significantly different from the full model (*P* = 0.11). Individual ID explained less than 0.001% of the variance and was therefore removed from the model. Bumblebees that developed at 27 °C visited flowers for significantly longer than those in 33 °C (*P* = 0.006, *r*^2^ = 0.05; Fig. 1A) and this was not affected by colony origin (*P* = 0.11), although visiting time tended to be lower for workers from 33 °C colonies.

The model that best explained the variation in visiting time of *Campanula persicifolia* (*n* = 88) included temperature treatment as a fixed factor, and individual ID as a random factor (next best model ΔAICc 1.99, Supplementary Table S3–S4 for details of the model), although it was not significantly different from the full model (*P* = 0.9). Visiting time was significantly higher for bumblebees that developed at 27 °C (*P* = 0.005, *r*^2^ = 0.18; Fig. 1B). The random factor, individual ID, explained 53.6% of the variance that remained in the residuals.

### Foraging behavior—visiting rate

The model that best explained the variation in visiting rate of *Borago officinalis* (*n* = 82) included temperature treatment as a fixed factor, and individual ID as a random factor (next best model ΔAICc 1.7, Supplementary Tables S5–S6 for details of the model). However, it was not significantly different from the full model (*P* = 0.87). We did not observe any significant impact of temperature treatment on visiting rate (*P* = 0.75, *r*^2^ = 0.01; Fig. 1C). The random factor individual ID explained 29% of the variance that remained in the residuals.

The model that best explained the variation in visiting rate of *Campanula persicifolia* (*n* = 66) included temperature treatment as a fixed factor and individual ID as a random factor (next best model ΔAICc 1.76, Supplementary Table S7–S8 for details of the model), although it was not significantly different from the full model (*P* = 0.33). We observed a significant impact of temperature on visiting rate (*P* < 0.001, *r*^2^ = 0.2; Fig. 1D). The random factor individual ID explained 32.9% of the variance that remained in the residuals.

### Foraging behavior—number of foraging trips

**Figure 2:**
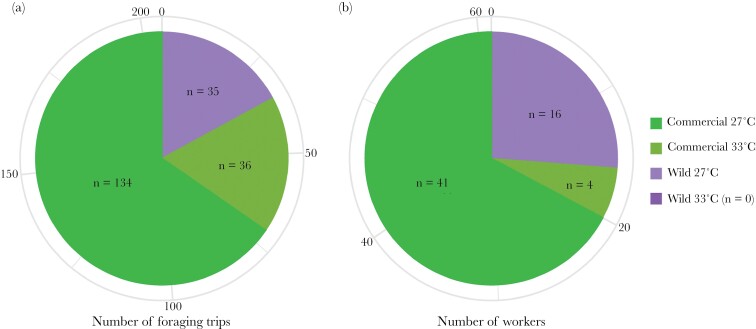
(A) Total number of foraging trips per temperature and colony origin. (B) Total number of workers performing the foraging trips per temperature and colony origin. No colors corresponding to the wild colonies reared at 33 °C can be found on the pie chart because no workers from this treatment performed foraging trips.

We first counted the total number of foraging trips per temperature treatment and per colony origin (Fig. 2A). In total, 41 workers from the commercial 27 °C colonies performed 134 trips, 16 workers from the wild 27 °C colonies performed 35 trips, 4 workers from commercial 33 °C colonies performed 36 trips, while no foraging trips were recorded for workers from wild 33 °C colonies (Fig. 2B). Thus, 82.4% of the recorded foraging trips were performed by workers from the 27 °C treatment.

To assess the effect of rearing temperature on the number of foraging trips from the colony to flowers at the individual level, we recorded the number of trips per individual, per day, for each temperature treatment. The model that best explained the number of trips per worker for *Borago officinalis* (*n* = 68) included temperature treatment as a fixed factor, and individual ID as a random factor (next best model ΔAICc 1.96, Supplementary Table S9 for details of the model), although it was not significantly different from the full model (*P* = 0.84). We observed that the average number of foraging trips performed by a worker at 33 °C was higher than for those reared at 27 °C (*P* < 0.001, *r*^2^ = 0.27; Supplementary Fig. S1). However, as very few workers from the 33 °C treatment left the colonies; the foraging trips were performed by the same few workers. As very few workers from 33 °C foraged on *Campanula persicifolia*, we were not able to assess the number of foraging trips on this plant species.

### Foraging behavior—foraging time

To assess the effect of rearing temperatures on foraging time, we recorded the time spent by each worker outside of the colony for each day. The model that best explained foraging time for *Borago officinalis* (*n* = 134) included temperature as a fixed factor and individual ID as a random factor (next best model ΔAICc 1.03, Supplementary Table S10–S11 for details of the model), although it was not significantly different from the full model (*P* = 0.325). Rearing temperature had a marginally significant effect on foraging time—bumblebees reared at 27 °C tended to spend more time out of the hive on foraging trips (*P* = 0.029, *r*^2^ = 0.04; Supplementary Fig. S2). The random factor individual ID explained 25.7% of the variance that remained in the residuals. As very few workers from 33 °C foraged on *Campanula persicifolia*, we were not able to assess the foraging time on this plant species.

### Morphological traits

**Figure 3:**
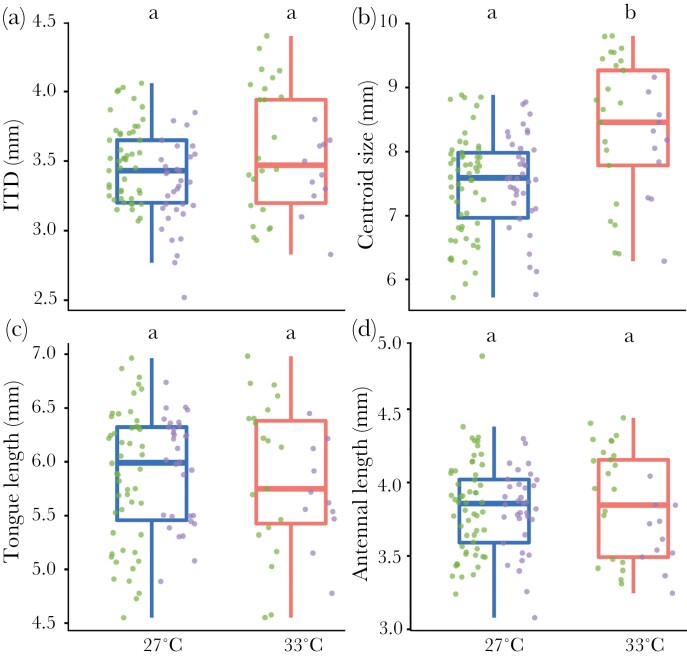
Impact of developmental temperature on bumblebee morphological traits. (A) ITD (mm), (B) centroid size (mm), (C) antennal length (mm), and (D) tongue length (mm). Letters at the top of the boxplots indicate significant differences when the letters are different. Green dots indicate specimens from commercial colonies, purple dots indicate specimens from wild colonies.

**Figure 4:**
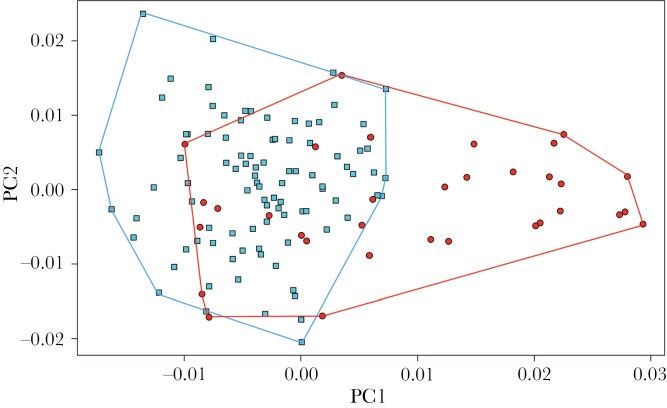
Ordination of the workers reared at the two developmental temperatures along the two first axes of a principal component analysis based on wing shape. Red points represent data from workers that developed in 33 °C, blue points represent data from workers that developed in 27 °C. Squares represent workers from commercial colonies, circles represent workers from wild colonies.

Body size, as measured by ITD was not significantly affected by colony origin (*P* = 0.09), temperature treatment (*P* = 0.21; Fig. 3A), or the interaction between these variables (*P* = 0.96) although, overall, the workers from wild colonies tended to be smaller than those from commercial colonies (*P* = 0.09). Workers reared at 33 °C had significantly larger wings than workers reared at 27 °C (*P* < 0.001; *r*^2^ = 0.21; Fig. 3B), but neither colony origin (*P* = 0.16) nor the interaction between temperature and colony origin had a significant effect on wing size (*P* = 0.12). Tongue length was not significantly affected by temperature (*P* = 0.7; Fig. 3C), colony origin (*P* = 0.32) or their interaction (*P* = 0.22). For ITD, wing size and tongue length, we removed colony ID from the model as it was explaining less than 0.001% of the variance that remained in the residuals. Neither temperature (*P* = 0.33; Fig. 3D) nor the interaction between temperature and colony origin (*P* = 0.12) had a significant impact on antennal length, but workers from wild colonies had significantly shorter antennae (*P* = 0.03, *r*^2^= 0.06). The random factor colony ID explained 2.1% of the variance that remained in the residuals. Finally, wing shape was significantly impacted by temperature (*P* = 0.001; Fig. 4), colony origin (*P* = 0.001) and the random factor “colony ID” (*P* = 0.001), but not by the interaction between colony origin and temperature (*P* = 0.5). All model details can be found in Supplementary Table S12–S17.

### Colony development

In total, the commercial colonies produced 1078 workers, with 722 from the 27 °C treatment and 356 workers from the 33 °C treatment. The wild colonies produced fewer individuals, with 275 workers in total—144 from the 27 °C treatment and 131 from the 33 °C treatment (Supplementary Fig. S3).

## DISCUSSION

While it is well known that global warming can lead to a phenological mismatch between plants and pollinators (Kehrberger and Holzschuh 2019; Kudo and Cooper 2019), less is known about how heat stress affects the pollination activity of insects. The main goal of this study was to begin to address this knowledge gap by assessing if high developmental temperatures have the potential to affect the foraging capabilities of bumblebees. Exposure to 33 °C during development led to a drastic reduction in the number of workers performing foraging trips and the total number of foraging trips. The few workers that foraged after developing in colonies that were exposed to 33 °C had a higher visiting rate and number of foraging trips per day per worker but a lower visiting time and foraging time with respect to the workers from colonies that were exposed the more optimal 27 °C treatment. Moreover, we found that high developmental temperatures affected both wing size and shape, and that the number of workers produced at 33°C was lower (722 workers produced at 27 °C versus 356 at 33 °C). This was particularly the case among commercial colonies, which produced 381 and 341 workers at 27 °C, while those in the 33 °C treatment produced only half this number (180 and 176 workers). Although we only have a limited number of data points (8 colonies), this result provides some indication that extended exposure to elevated temperatures may be stressful enough to sharply decrease the number of workers produced. The elevated temperatures could thus have a direct impact during the development, on the colony growth and the genesis of morphological traits, but also a delayed impact on the foraging behavior, which could amplify the direct effects on colony fitness.

Rearing temperature had several striking effects on foraging behavior. Firstly, the workers in colonies from the 33 °C treatment performed many fewer foraging trips than workers from the colonies in the 27 °C treatment (*n* = 4 vs *n* = 57, respectively; around 93% fewer from 33 °C than from 27 °C). Secondly, workers reared at 33 °C performed many fewer foraging trips in total compared with those reared at 27 °C (*n* = 36 vs *n* = 169, respectively, around 79% fewer from 33 °C than from 27 °C). This result may be partly explained by differences in worker production—colonies in the 33 °C treatment produced around 40% fewer workers than those in the 27 °C treatment—although this would still not explain these results fully because we observed 79% fewer foraging trips in total. Another possible explanation is that the changes in the wing morphology that we observed in workers that developed in the different temperatures, which could impact flight and foraging performance (DeVries et al. 2010; Foster and Cartar 2011, see discussion below for further details).

Converse to the expectations of the TSR, we did not observe a decrease in worker body size (or any other measured morphological features) at the elevated rearing temperature. One of the potential mechanisms for the TSR is that, at higher temperatures, growth rate is faster and development time decreases, leading to smaller adults (Atkinson 1994; Angilletta and Dunham 2003). Due to their partial endothermy and sociality, bumblebee colonies may buffer this effect by fanning. This is particularly true for bumblebee species with large colonies, like *B. terrestris*, but the advantage of sociality is probably weaker in species that have smaller colonies (i.e., only dozens of individuals for some species; Cueva del Castillo et al. 2015), where we would expect a stronger impact of the temperature on body size. As fanning costs energy, this could be why the colonies at higher temperatures produced fewer individuals: energy spent maintaining a constant temperature cannot be spent on producing more individuals. It is nonetheless interesting to note that *B. terrestris* is a rare case of a European bumblebee that is originated from warm Mediterranean climates (Rasmont et al. 2015), conversely to most of bumblebee species that have a boreal or a temperate origin. This is even more concerning: we may have expected that this species would be more robust to warmer developmental temperatures. The effects of elevated developmental temperatures on boreal species could thus be even more deleterious.

Both wing size and shape were affected by development under heat stress, which is consistent with the results of an earlier study on male bumblebees (Gérard et al. 2018a). Wings of workers reared at 33 °C were broader, particularly the cell at the basis of the wing (i.e., second cubital cell), and slightly longer (Fig. 4, Supplementary Fig. S4). Longer and slender wings are better adapted for long distance flight, while broader but shorter wings are better for maneuverability (Betts and Wooton 1988; DeVries et al. 2010). Maneuverability is probably more important for floral resource collection and entering back in the colony, particularly in the framework of our experiment where the room prevented long distance flight. It is interesting that we observed both longer and broader wings in workers reared at 33 °C, as this suggests that these individuals may be better adapted to longer foraging flights, although more detailed flight control (Baird 2020) and field investigations would be necessary to determine if this is indeed the case. Interestingly, we observed that body size and wings were not similarly affected by developmental temperature. As only the wing morphology changed significantly, we can hypothesize that the resilience of these two morphological traits to temperature are different, and that their variation is not isometric. The non-isometric relationship of two morphological traits has already been observed (McDonald et al. 2018), and it is known can it may also impact foraging behavior (Riveros and Gronenberg 2010; Peters et al. 2016).

In addition to changes in wing morphology and potential effects on colony development, there are several additional ways in which exposure to elevated temperatures during development could affect bumblebee pollination behavior, even potentially before any morphological changes occur. For example, heat stress may affect processes of memory and cognition, capabilities that are crucial for efficient foraging. When temperatures exceed their optimal developmental temperature, the ability of honeybees to correctly perform the waggle dance is impaired (Tautz et al. 2003). In bumblebees, stressful temperatures can impact short term memory (Jones et al. 2013; Gérard et al., 2022), as well as the development of some brain structures such as the mushroom bodies (Groh et al. 2004, 2006). We hypothesize that elevated developmental temperatures lead to cognition and memory impairments in bumblebees that, in turn, stunned them and affected flight performance and foraging efficiency. Elevated temperatures are also likely to cause workers to allocate a large amount of their energy budget to colony thermoregulation, reducing the amount of energy they can spend foraging. It is possible that workers reared in 33 °C were compensating for this by increasing their visiting rate: the few workers that left their colonies visited more flowers per minute and performed more foraging trips per worker and per day. Conversely, workers from 27 °C spent longer outside the colony and took more time to collect resources on each flower. Richman et al. (2020) found that, at low elevations, visiting rate can increase with temperature in alpine pollinator communities, which can sometimes increase pollination efficiency. However, visiting rate is not always directly linked to pollination efficiency (Ne’eman et al. 2009). Visiting rate can only be a reasonable proxy of pollination efficiency if the variation of the visiting rate is larger than the variation of pollination efficiency per visit (Sahli and Conner 2006; Benadi and Pauw 2018). Indeed, pollination efficiency is more affected by the amount of viable pollen deposited and studies on this topic tend to show that visiting rate is not a direct indicator of pollination efficiency (King et al. 2013).

In addition to the parameters we measured, plant–pollinator interactions can also be threatened by global warming from the plant perspective. Some plant phenotypic changes can occur due to high temperature and drought, which can also alter bumblebee behavior (Höfer et al. 2021). For example, a decrease of corolla length or floral size can lead to a lower visiting rate (Burkle and Runyon 2016; Gallagher and Campbell 2017; Descamps et al. 2021). Additionally, the heat stress we exposed bees to here was only applied during development and not during the plant–pollinator experiments. A recent study highlighted that flight performance (measured as flight duration and flight speed) is dependent on the ambient temperature (Kenna et al. 2021). The highest flight performances were around 25 °C and decreased as the ambient temperature approached 30 °C. The results of this study, combined with our results, suggest that during prolonged heatwaves, not only temperatures during the development would impact bumblebee life cycle, but also high ambient temperature during their flight, which could lead to synergetic effects and have an even worse impact on foraging behavior than what we observed here. While we showed for the first time that foraging behavior can be strongly impacted by heat stress in bumblebees, further studies should assess the impact of increased rearing temperatures on pollination effectiveness by evaluating, for example, single visit pollen deposit as well as seed- and fruit-set. It could help in understanding if the differences we observed in foraging behavior, morphology and colony development ultimately impact pollination efficiency.

## Supplementary Material

arac045_suppl_Supplementary_Material
