# Supplementary material for: Exposure to elevated temperature during development affects bumblebee foraging behavior
Source: Behav Ecol. 2022 Jun 3;33(4):816–24. doi: 10.1093/beheco/arac045 (PMC9262166; doi:10.1093/beheco/arac045)
Supplement: arac045_suppl_Supplementary_Material [file arac045_suppl_supplementary_material.docx]

**Supplementary Information for**

**Exposure to elevated temperature during development affects bumblebee foraging behaviour**

Maxence Gérard^1^*, Bérénice Cariou^1,2^, Maxime Henrion^1,3^, Charlotte Descamps^4^, Emily Baird^1^

^1^ INSECT Lab, Division of Functional Morphology, Department of Zoology, Stockholm University, Svante Arrhenius väg 18b, 11418, Stockholm, Sweden

^2^ Sorbonne Université, Faculté des Sciences et Ingénierie, 5 place Jussieu, 75005, Paris, France.

^3^ Ecole Normale Supérieure de Lyon, 15 parvis René Descartes, Lyon, France

^4^ Earth and Life Institute-Agronomy, UCLouvain, Croix du Sud 2, box L7.05.14, 1348 Louvain-la-Neuve, Belgium

* Corresponding author: [maxence.gerard@zoologi.su.se](mailto:maxence.gerard@zoologi.su.se) ; ORCID : <https://orcid.org/0000-0002-2485-0662>


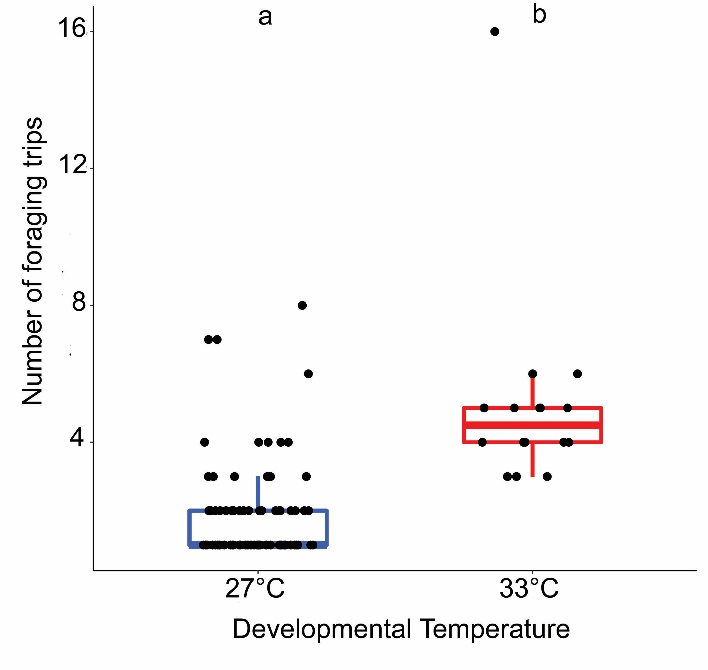


**Figure S1.** Impact of developmental temperature on bumblebee foraging behavior. Number of foraging trips for *Borago* (n = 96).


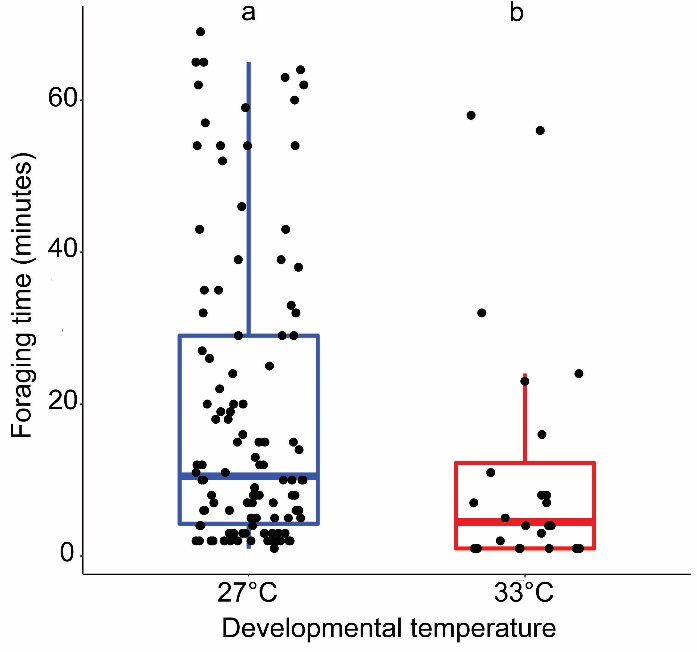


**Figure S2.** Impact of developmental temperature on bumblebee foraging behavior. Foraging time for *Borago* (n = 134). Letters at the top of the boxplots indicate significant differences when the letters are different.


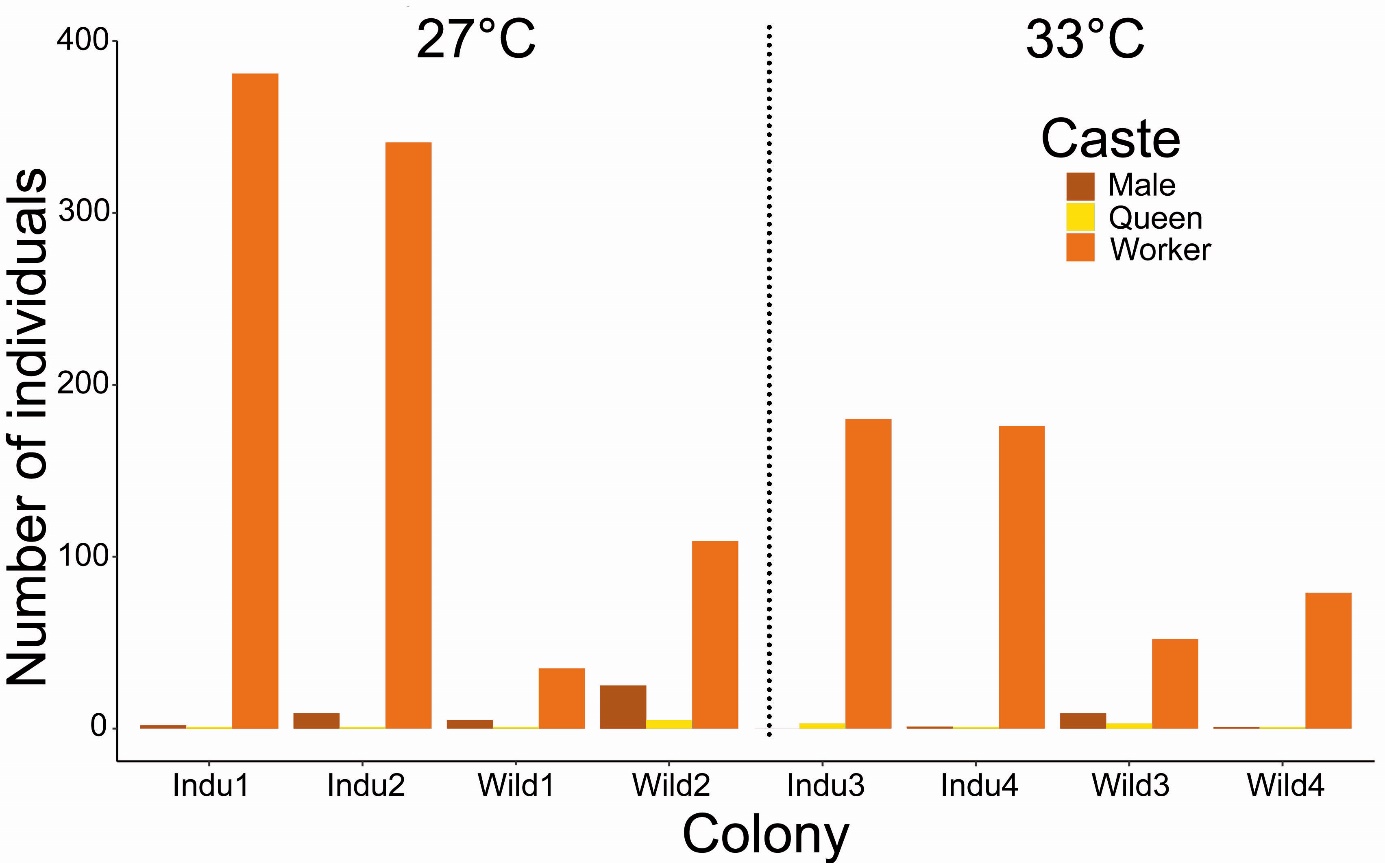


**Figure S3.** Number of individuals produced in each colony, depending on the caste, the colony origin and the environmental temperature (27°C or 33°C). Colonies Indu 1-4 are industrial colonies; colonies Wild1-4 are colonies from wild-caught queens. Colony production was significantly affected by colony origin (p = 0.029) but not temperature (p = 0.686).


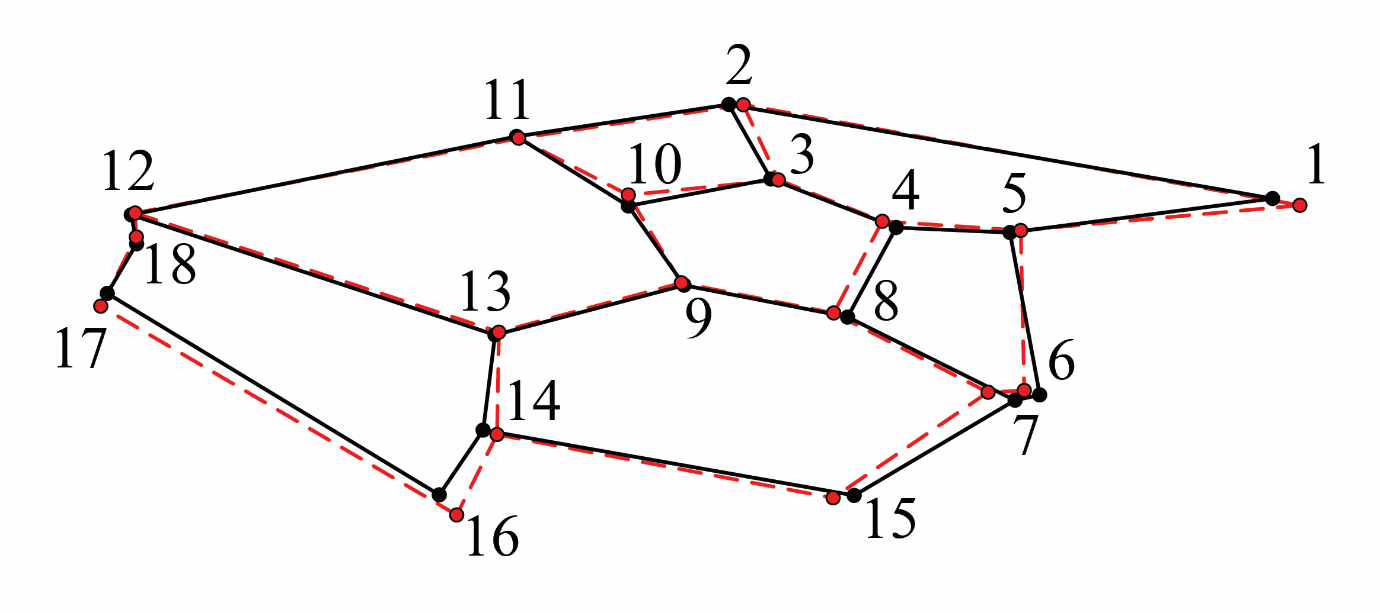


**Figure S4.** Mean wing shape differences between bumblebees developed in 27°C (in black) and 33°C (in red). The numbers represent the landmark ID.

**Table S1.** Dataset for the morphological traits per colony

| **Colony ID** | **Colony Origin** | **Temperature (**°**C)** | **N** |
| --- | --- | --- | --- |
| A | Industrial | 27 | 25 |
| B | Industrial | 27 | 23 |
| C | Wild | 27 | 11 |
| D | Wild | 27 | 25 |
| E | Industrial | 33 | 8 |
| F | Industrial | 33 | 15 |
| G | Wild | 33 | 4 |
| H | Wild | 33 | 7 |

| **Table S2.**  Linear model output of the best model (lowest AICc) to predict visiting time on *Borago officinalis*. | | | | |
| --- | --- | --- | --- | --- |
|  | Visiting time | | |  |
| Predictors | Estimates | Std Error | p |  |
| (Intercept) | 1.763 | 0.061 | <0.001 |  |
| Temperature | 0.203 | 0.072 | 0.006 |  |
| Colony origin | -0.106 | 0.066 | 0.109 |  |
| DF | 166 | | |  |
| Multiple R-squared | 0.05 | | |  |
| Adjusted R-squared | 0.04 | | |  |
| Model structure is log(VisitingTime +1) ~ Temperature + Colony origin | | | | |

| **Table S3.**  Linear mixed model output of the best model (lowest AICc) to predict visiting time on *Campanula persicifolia*. | | | | | |  |  |
| --- | --- | --- | --- | --- | --- | --- | --- |
|  |  | | Visiting time | | | |  |
| Predictors | Estimates | Std Error | | df | p | |  |
| (Intercept) | 4.411 | 0.784 | | 27.245 | <0.001 | |  |
| Temperature | 2.288 | 0.918 | | 27.911 | 0.004 | |  |
| R-squared fixed effect |  | | 0.18 | | | |  |
| R-squared random effect |  | | 0.39 | | | |  |
| Model structure is VisitingTime ~ Temperature + (1 \| ID) | | | | | |  |  |

| **Table S4.**  Proportion of variance explained by the random factor in the best linear mixed model predicting visiting time on *Campanula persicifolia*. | | | | |
| --- | --- | --- | --- | --- |
|  | Random effects | | |  |
| Groups | Variance | Std. Deviation |  |  |
| Individual ID | 4.275 | 2.068 |  |  |
| Residual | 3.696 | 1.923 |  |  |
| # Observations | 88 | | |  |
| # Individual ID | 41 | | |  |

| **Table S5.**  Linear mixed model output of the best model (lowest AICc) to predict visiting rate on *Borago officinalis*. | | | | | |  |  |
| --- | --- | --- | --- | --- | --- | --- | --- |
|  |  | | Visiting rate | | | |  |
| Predictors | Estimates | Std Error | | df | p | |  |
| (Intercept) | 0.893 | 0.246 | | 10.774 | 0.004 | |  |
| Temperature | -0.088 | 0.265 | | 12.023 | 0.745 | |  |
| R-squared fixed effect |  | | 0.01 | | | |  |
| R-squared random effect |  | | 0.27 | | | |  |
| Model structure is VisitingRate ~ Temperature + (1 \| ID) | | | | | |  |  |

| **Table S6.**  Proportion of variance explained by the random factor in the best linear mixed model predicting visiting rate on *Borago officinalis*. | | | | |
| --- | --- | --- | --- | --- |
|  | Random effects | | |  |
| Groups | Variance | Std. Deviation |  |  |
| Individual ID | 0.131 | 0.361 |  |  |
| Residual | 0.296 | 0.544 |  |  |
| # Observations | 82 | | |  |
| # Individual ID | 32 | | |  |

| **Table S7.**  Linear mixed model output of the best model (lowest AICc) to predict visiting rate on *Campanula persicifolia*. | | | | | |  |  |
| --- | --- | --- | --- | --- | --- | --- | --- |
|  |  | | Visiting rate | | | |  |
| Predictors | Estimates | Std Error | | df | p | |  |
| (Intercept) | 5.454 | 0.6 | | 22.885 | <0.001 | |  |
| Temperature | -3.633 | 0.655 | | 25.552 | <0.001 | |  |
| R-squared fixed effect |  | | 0.2 | | | |  |
| R-squared random effect |  | | 0.29 | | | |  |
| Model structure is VisitingRate ~ Temperature + (1 \| ID) | | | | | |  |  |

| **Table S8.**  Proportion of variance explained by the random factor in the best linear mixed model predicting visiting rate on *Campanula persicifolia*. | | | | |
| --- | --- | --- | --- | --- |
|  | Random effects | | |  |
| Groups | Variance | Std. Deviation |  |  |
| Individual ID | 0.829 | 0.911 |  |  |
| Residual | 1.696 | 1.302 |  |  |
| # Observations | 66 | | |  |
| # Individual ID | 35 | | |  |

| **Table S9.**  Linear mixed model output of the best model (lowest AICc) to predict the number of foraging trips on *Borago officinalis*. | | | | | |  |  |
| --- | --- | --- | --- | --- | --- | --- | --- |
|  |  | | Number of Foraging trips | | | |  |
| Predictors | Estimates | Std Error | | p |  |  |  |
| (Intercept) | 1.579 | 0.24 | | <0.001 |  |  |  |
| Temperature | -1.071 | 0.268 | | <0.001 |  |  |  |
| R-squared fixed effect |  | | 0.27 | | | |  |
| R-squared random effect |  | | 0.29 | | | |  |
| Model structure is NbForagingTrips ~ Temperature + (1 \| ID) | | | | | |  |  |

| **Table S10.**  Linear mixed model output of the best model (lowest AICc) to predict foraging time on *Borago officinalis*. | | | | | |  |  |
| --- | --- | --- | --- | --- | --- | --- | --- |
|  |  | | Foraging time | | | |  |
| Predictors | Estimates | Std Error | | df | p | |  |
| (Intercept) | 1.424 | 0.423 | | 15.705 | 0.004 | |  |
| Temperature | 1.082 | 0.448 | | 16.956 | 0.027 | |  |
| R-squared fixed effect |  | | 0.04 | | | |  |
| R-squared random effect |  | | 0.24 | | | |  |
| Model structure is Foraging time ~ Temperature + (1 \| ID) | | | | | |  |  |

| **Table S11.**  Proportion of variance explained by the random factor in the best linear mixed model predicting foraging time on *Borago officinalis*. | | | | |
| --- | --- | --- | --- | --- |
|  | Random effects | | |  |
| Groups | Variance | Std. Deviation |  |  |
| Individual ID | 0.33 | 0.575 |  |  |
| Residual | 1.115 | 1.056 |  |  |
| # Observations | 134 | | |  |
| # Individual ID | 43 | | |  |

| **Table S12.**  Linear model output of the model to predict ITD | | | | |
| --- | --- | --- | --- | --- |
|  | ITD | | |  |
| Predictors | Estimates | Std Error | p |  |
| (Intercept) | 3.614 | 0.07 | <0.001 |  |
| Temperature | -0.107 | 0.085 | 0.212 |  |
| Colony origin | -0.213 | 0.123 | 0.086 |  |
| Temperature:Colony origin | 0.007 | 0.145 | 0.959 |  |
| DF | 111 | | |  |
| R-squared | 0.11 | | |  |
| Model structure is ITD ~ Temperature*Colony origin | | | | |

| **Table S13.**  Linear model output of the model to predict wing size. | | | | |
| --- | --- | --- | --- | --- |
|  | Centroid size | | |  |
| Predictors | Estimates | Std Error | p |  |
| (Intercept) | 0.118 | 0.003 | <0.001 |  |
| Temperature | 0.017 | 0.003 | <0.001 |  |
| Colony origin | 0.007 | 0.005 | 0.155 |  |
| Temperature:Colony origin | -0.009 | 0.006 | 0.122 |  |
| DF | 126 | | |  |
| R-squared | 0.21 | | |  |
| Model structure is CentroidSize ~ Temperature*Colony origin | | | | |

| **Table S14.**  Linear model output of the model to predict tongue length | | | | |
| --- | --- | --- | --- | --- |
|  | Tongue | | |  |
| Predictors | Estimates | Std Error | p |  |
| (Intercept) | 0.169 | 0.004 | <0.001 |  |
| Temperature | 0.002 | 0.005 | 0.703 |  |
| Colony origin | 0.007 | 0.007 | 0.319 |  |
| Temperature:Colony origin | -0.009 | 0.008 | 0.222 |  |
| DF | 112 | | |  |
| R-squared | 0.02 | | |  |
| Model structure is Tongue ~ Temperature*Colony origin | | | | |

| **Table S15.**  Linear mixed model output of the model to predict antennae length. | | | | | |  |  |
| --- | --- | --- | --- | --- | --- | --- | --- |
|  |  | | Antenna | | | |  |
| Predictors | Estimates | Std Error | | df | p | |  |
| (Intercept) | 3.945 | 0.076 | | 6.727 | <0.001 | |  |
| Temperature | -0.103 | 0.093 | | 4.156 | 0.327 | |  |
| Colony Origin | -0.308 | 0.126 | | 12.958 | 0.03 | |  |
| Temperature:ColonyOrigin | 0.267 | 0,151 | | 7.09 | 0.12 | |  |
| R-squared fixed effect |  | | 0.06 | | | |  |
| R-squared random effect |  | | 0.2 | | | |  |
| Model structure is Antennae ~ Temperature*ColonyOrigin + (1 \| ID) | | | | | |  |  |

| **Table S16.**  Proportion of variance explained by the random factor in the linear mixed model predicting antenna length. | | | | |
| --- | --- | --- | --- | --- |
|  | Random effects | | |  |
| Groups | Variance | Std. Deviation |  |  |
| Colony ID | 0.002 | 0.047 |  |  |
| Residual | 0.1 | 0.314 |  |  |
| # Observations | 124 | | |  |
| # Colony ID | 8 | | |  |

| **Table S17.**  Linear model with randomized residual (lm.rrpp) output to predict wing shape | | | | |
| --- | --- | --- | --- | --- |
|  | Wing shape | | |  |
| Predictors | Sum of squares | R-squared | p |  |
| Temperature | 0.0057 | 0.1016 | 0.001 |  |
| Colony Origin | 0.0015 | 0.0258 | 0.001 |  |
| Temperature:Colony Origin | 0.0025 | 0.0451 | 0.514 |  |
| Temperature: ColonyID | 0.0102 | 0.182 | 0.001 |  |
| Residuals | 0.0363 | 0.6455 |  |  |
| # Observations | 127 | | |  |
| R-squared | 0.355 | | |  |
| Model structure is Wing shape ~ Temperature * Type + Temperature:Colony ID | | | | |
